# TSPAN4 controls vascular smooth muscle cell phenotypic switching and intimal hyperplasia by targeting TPM1-regulated cytoskeletal organization

**DOI:** 10.1042/CS20255833

**Published:** 2025-10-08

**Authors:** Shengbiao Li, Kexin Chen, Yi Zhang, Yang Yu, Tianyi Zhang, Donghui Jiang, Mi Li, Shubo Fu, Ji chen, Jiapan Li, Jingyan Yi, Rong Li, Gan Qiao, Jianguo Feng, Jun Jiang, Qiong yuan, Chunxiang Zhang

**Affiliations:** 1 School of Basic Medical Sciences, Nucleic Acid Medicine of Luzhou Key Laboratory, Basic Medicine Research Innovation Center for Cardiometabolic Diseases, Southwest Medical University, Luzhou, Sichuan, 646000, China; 2 Department of Cardiology, West China Hospital, Sichuan University, Chengdu, 610041, China; 3 Department of Cardiology, The Affiliated Hospital of Southwest Medical University, Nucleic Acid Medicine of Luzhou Key Laboratory, Key Laboratory of Medical Electrophysiology, Ministry of Education, Institute of Cardiovascular Research, Southwest Medical University, Luzhou, Sichuan, 646000, China; 4 Basic Medicine Research Innovation Center for Cardiometabolic Diseases, Ministry of Education; Luzhou Municipal Key Laboratory of Thrombosis and Vascular Biology; Laboratory for Cardiovascular Pharmacology, Department of Pharmacology, School of Pharmacy, Southwest Medical University, Luzhou, 646000, China; 5 Department of Pharmacology, School of Pharmacy, Nucleic Acid Medicine of Luzhou Key Laboratory, Central Nervous System Drug Key Laboratory of Sichuan Province, Southwest Medical University, Luzhou, Sichuan, 646000, China; 6 Anesthesiology and Critical Care Medicine Key Laboratory of Luzhou, the Affiliated Hospital, Southwest Medical University, Luzhou, Sichuan Province, 646000, China; 7 Department of General Surgery (Thyroid Surgery), the Affiliated Hospital of Southwest Medical University, Luzhou, Sichuan, 646000, China

**Keywords:** actin cytoskeleton, intimal hyperplasia, phenotypic switching, tropomyosin-1, TSPAN4, vascular smooth muscle cell

## Abstract

Vascular smooth muscle cell (VSMC) phenotypic switching, followed by enhanced proliferation and migration, is a key event in the development of intimal hyperplasia in diverse vascular diseases. While tetraspanin 4 (TSPAN4) is known to be expressed in the vasculature, its function in VSMC phenotypic switching and vascular disease is currently unknown. Here, we investigated the role of TSPAN4 using an *in vitro* model of platelet-derived growth factor BB (PDGF-BB)-induced phenotypic switching and an in *vivo* carotid artery ligation model in wildtype and TSPAN4-deficient mice. Our experiments, including EdU assays, Transwell assays, western blot analysis, and immunoprecipitation, revealed that TSPAN4 expression is elevated in human atherosclerotic arteries, ligated mouse carotid arteries, and PDGF-BB-stimulated VSMCs. Additionally, TSPAN4 overexpression promoted the switch from a contractile to a synthetic phenotype, accompanied by enhanced VSMC proliferation and migration. Conversely, TSPAN4 knockdown inhibited these effects, suppressing PDGF-BB-induced phenotypic switching. Mechanistically, TSPAN4 was found to interact with and influence the expression and localization of tropomyosin-1 (TPM1). This, in turn, affected cytoskeletal organization, ultimately driving phenotypic switching and functional alterations in VSMCs. Finally, we demonstrated that TSPAN4 deficiency in mice attenuated vascular neointimal formation following carotid artery ligation. These findings suggested that TSPAN4 is a promising novel therapeutic target for vascular remodeling and proliferative vascular diseases.

## Introduction

Vascular smooth muscle cells (VSMCs) are critical components of the vascular wall and play a crucial role in vascular remodeling and the development of vascular diseases in response to diverse stimuli. VSMCs exhibit strong plasticity and exist in two main phenotypic states: contractile and synthetic. Under physiological conditions, VSMCs typically remain in a contractile state, where their primary function is to maintain vascular contractile function and regulate vascular tone, thereby controlling systemic and local blood flow [1]. However, in response to certain regulatory factors such as platelet-derived growth factor BB (PDGF-BB) or pathological stimuli, VSMCs can reversibly switch from this contractile phenotype to synthetic one, known as phenotypic switching. The synthetic phenotype is characterized by enhanced proliferation and migration, increased synthesis of extracellular matrix, decreased expression of cell-specific contractile marker proteins, and up-regulated expression of adhesion glycoproteins such as osteopontin (OPN) and epiregulin [2,3]. Synthetic VSMCs migrate toward the endothelium, where they cause neointimal hyperplasia with inflammatory features, ultimately resulting in vascular diseases such as atherosclerosis, hypertension, restenosis after angioplasty, diabetic vascular complications, and transplantation arteriopathy [4–7]. Therefore, therapeutic strategies targeting VSMC phenotypic switching could be effective in preventing and treating these conditions.

The mechanisms underlying VSMC phenotypic switching are still unclear. However, it is known that many signaling pathways and transcription factors are involved in these phenotypic changes [1,8–10]. At the cell structure level, the molecular alterations that occur during phenotypic switching finally elicit cytoskeletal changes in VSMCs, followed by their functional modulation [1,8–10]. Stress fibers are primarily composed of actin filaments and are essential components of the cytoskeleton. A key feature in VSMC phenotypic switching is stress fiber rearrangement [11–13]. Synthetic VSMCs typically exhibit fewer, shorter, and/or weaker stress fibers than their contractile counterparts. An increasing number of studies have indicated that these cytoskeletal changes, referred to as cytoskeletal dynamics, make a substantial contribution to the development of vascular diseases, although the exact molecular mechanisms remain unclear [11,13,14].

The tetraspanin family of proteins comprises transmembrane proteins with a unique ability to form complexes on cell membranes, thereby regulating a range of biological processes. Research on tetraspanins has garnered significant attention over recent years [15–17]. They regulate key cellular processes such as cell adhesion, migration, and fusion by interacting with cell adhesion proteins, growth factor receptors, and members of the Ig superfamily, among other membrane proteins [18,19]. These properties make tetraspanins essential for cell survival, signaling, and immune responses. Several studies, including one by our group, have identified possible correlations between the expression levels of the tetraspanin family members CD151 and CD9 and vascular disease [20–24].

Tetraspanin 4 (TSPAN4), as a member of the tetraspanin family, has received increasing attention for its important cellular functions. TSPAN4 is expressed in diverse cell types and is involved in a wide range of biological processes [25,26]. Recent studies have identified TSPAN4 as a key regulator of a newly discovered organelle called the migrasome [27,28], which is closely associated with cellular functions such as migration and other biological activities [29–31]. In addition to its role in normal physiological processes, the aberrant expression of TSPAN4 has been strongly implicated in the onset and progression of several diseases. A few bioinformatic studies have also identified a potential association between TSPAN4 and vascular diseases [24,25,32]. However, whether there is a relationship between TSPAN4 and the phenotypic transition of VSMCs is currently unknown, as is its precise role in vascular disease.

Here, we first found that TSPAN4 expression is elevated in vessels exhibiting neointima formation and that TSPAN4 deficiency in VSMCs maintains these cells in a contractile state. Subsequently, we sought to determine the role of TSPAN4 in VSMC phenotypic switching and vascular disease, as well as investigate the underlying mechanisms both *in vitro* and *in vivo*.

## Materials and methods

### Animals

Twelve-week-old male C57BL/6J wildtype (WT) mice and TSPAN4-deficient (*Tspan4* ^−/−^) mice, both on a C57BL/6J genetic background, were used for this study. The TSPAN4 knockout mice were obtained from Cyagen Biotechnology Co., Ltd., China. All the mice were housed in SPF-grade animal facilities and acclimatized to the experimental environment for one week before commencing the corresponding experiments. Sterile food, water, and bedding were provided and regularly replenished. Mice were killed by inhalation of an overdose of isoflurane (5% isoflurane in 100% oxygen), followed by cervical dislocation to ensure end of life. The animals were monitored throughout the procedure to confirm complete loss of consciousness and the absence of reflexes, ensuring a humane and ethical end of life.

### Carotid artery ligation

The necks of mice were meticulously debrided, and body weight was recorded before surgery. Anesthesia was induced *via* the intraperitoneal injection of 3% sodium pentobarbital at a dose of 50 mg/kg. After immobilization, the mice were placed in a supine position with limbs and head secured, and the neck was fully exposed. The site of surgery was disinfected with iodine, after which a 1–1.5 cm midline incision was made. Under stereomicroscopic guidance, the muscle layer was gently separated, and the left common carotid artery was ligated using a 7–0 suture. Subsequently, the neck muscles and other tissues were carefully repositioned, sterilized with iodine, and the incision was sutured.

### Histological and morphometric analysis of the carotid arteries

Before the experiment, instruments such as spring scissors, forceps, and regular scissors were sterilized by autoclaving and drying. Mice were killed and positioned supine. The neck muscles and surrounding tissues were dissected to expose and remove the carotid arteries and aorta. Vessel tissues intended for protein and RNA extraction were snap-frozen in liquid nitrogen and stored at −80°C. For histological examination, tissues were fixed in 4% paraformaldehyde, dehydrated, embedded in OCT compound, frozen, sectioned, and stored at −20°C.

For hematoxylin and eosin (H&E) staining, frozen sections were thawed for 30 minutes, stained with hematoxylin for 3–5 minutes, dehydrated in 95% ethanol for 5 minutes, stained with eosin for 1 minute, dehydrated twice in absolute ethanol, 5 minutes each time, cleared in xylene for 5 minutes, mounted with neutral resin, and scanned using a digital slide scanner.

### Immunofluorescence

For mouse carotid artery tissue sections and cells, the following procedure was used: Tissue sections were thawed for 30 minutes, washed with PBS, outlined with a hydrophobic barrier pen, and treated with proteinase K. After another PBS wash, the samples were permeabilized, blocked, incubated with primary antibodies overnight, washed again, incubated with corresponding species-specific secondary antibodies, and imaged using a confocal microscope. For cells, after seeding into culture plates and treatment, the same steps were followed as for tissue sections after the washing stage. Actin-Tracker Red-Rhodamine (C2207S, Beyotime Biotechnology) was used for staining the cytoskeleton. Post-acquisition, images were analyzed using Image J software. To assess the spatial relationship among TSPAN4, F-actin, and TPM1 expression, immunofluorescence images were acquired using a Zeiss confocal laser scanning microscope under consistent acquisition settings. Images were analyzed with ZEN software (Carl Zeiss). For each experimental condition, at least four independent biological replicates were imaged, and from each replicate, 20 cells were randomly selected from a minimum of six different fields of view. The quantification of the mean fluorescence intensity in regions of interest (ROIs) and the generation of one-dimensional profile curves were performed using ZEN software and GraphPad Prism 9.0. Statistical analyses were conducted using one-way ANOVA followed by pairwise *t*-tests, with *P* values of <0.05 considered significant. The antibodies employed for immunofluorescence are listed in Supplementary Table S1.

### Cell culture

Human primary aortic smooth muscle cells (HASMCs) were procured from Zhejiang Meisen Cell Technology Company (Catalog No. CTCC-170-HUM) and were cultured in smooth muscle basal medium (Catalog No. CTCC-004-PriMed) supplemented with 10% FBS, 1% EGF, and 1% Penicillin-Streptomycin. The cells were maintained at a temperature of 37°C in an incubator with a humidified atmosphere composed of 95% air and 5% CO2. At approximately 70% confluence, the cells were trypsinized using 0.25% trypsin-EDTA solution.

### Wound healing assay

Cells were seeded into a 12-well plate and cultured to confluence. After 24 h of serum starvation, a cell-free zone was created by gently scratching the monolayer with a 200 μl pipette tip. Subsequently, the detached cells were removed by rinsing with PBS, and culture was continued in either complete medium or serum-free medium (for cells treated with PDGF-BB). The scratched area was immediately imaged under an Olympus CKX41 inverted microscope. After 24 h, a second set of images was acquired to assess cell migration into the wound area. The wound area in each well was quantified using ImageJ software, and the percentage of wound closure was calculated by comparing the size of the cell-free areas at 0 and 24 h.

### Transwell assay

Cultured VSMCs were trypsinized, centrifuged, and resuspended in complete medium. Next, 200 μl of the cell suspension was transferred to Transwell inserts placed in a 24-well plate, with the medium in the lower chamber containing 10% FBS. After 12 h of incubation, the cells in the inserts were fixed, stained, and imaged. Cells were counted in five fields of view per sample for statistical analysis.

### EdU incorporation assay

VSMCs were cultured on sterile cell slides in 24-well plates to 80% confluence. For PDGF-BB treatment, once the cells had reached confluence, the medium was replaced with serum-free medium, and the cells were incubated for an additional 48 h with or without PDGF-BB. To assess cell proliferation, a 1:500 dilution of a 10 mM EdU solution (EdU assay kit from Beyotime, Catalog No. C0078S) was prepared, resulting in a 2 × EdU working solution. This working solution was added to the 24-well plate containing the cultured cells, followed by incubation for 2 h. Subsequently, the cells were fixed, permeabilized, washed, and incubated with a Click reaction solution. Finally, the samples were sealed using an anti-fluorescence quenching mounting agent containing DAPI and imaged under a fluorescence microscope.

### Real-time polymerase chain reaction

Total RNA was extracted using TRIzol reagent (Life Technologies) and subsequently reverse-transcribed into cDNA using the ReverTra Ace qPCR RT Kit (Vazyme). Real-time fluorescence quantitative PCR (qPCR) was performed using the SYBR® Premix Ex TaqTM Kit (Thermo Fisher Scientific) on a QuantStudio 5 instrument (Thermo Fisher Scientific). The expression levels of target genes were normalized to that of *GAPDH* and calculated using the 2^-△△^Ct method. The primer sequences were as follows:

mus-TSPAN4 (F5′-TACCTCATGTTCGCCTTCAAC-3′, R5′-GATAAGGTGGCAAAGTTTCCCT-3′), mus-GAPDH (F5′-GAGAAACCTGCCAAGTATGATGAC-3′, R5′-AGAGTGGGAGTTGCTGTTGAAG-3′), homo-TSPAN4 (F5′-ACTTTGCCACCTTATCATCCTCAT -3′, R5′- TCTTCAGGTCTTGTTGGGCATAA-3′), and homo-GAPDH (F5′-GAGAAACCTGCCAAGTATGATGAC-3′, R5′-AGAGTGGGAGTTGCTGTTGAAG-3′).

### Western blot analysis

Proteins were extracted from cells or tissue using RIPA lysis buffer containing phosphoproteinase inhibitors and PMSF. Protein samples were separated by SDS-PAGE and transferred to nitrocellulose membranes using the Bio-Rad western blotting system. The membranes were blocked with 5% BSA or 5% nonfat dry milk in Tris-buffered saline containing 0.1% Tween 20 for 1 h at room temperature and then incubated with primary antibodies overnight at 4°C. Following incubation with the appropriate horseradish peroxidase-conjugated secondary antibodies, the blots were visualized and imaged using an ECL Kit (Epizyme) and the ChemiDoc Imaging System (Tianneng). Gel images were densitometrically analyzed using ImageJ software. Protein levels were normalized to β-actin (Proteintech) and the corresponding total protein content. The antibodies used for western blot are listed in Supplementary Table S1.

### Co-immunoprecipitation (co-IP)

VSMCs overexpressing TSPAN4 were harvested after washing with PBS and lysed in IP lysis buffer (150 mM KCl, 25 mM Tris, pH 7.4, 5 mM EDTA, 0.5 mM DTT, 0.5% NP-40) freshly supplemented with 100 U/ml RNase inhibitor and a protease inhibitor cocktail. All procedures were carried out on ice to preserve protein integrity. Cells were homogenized using a pre-chilled glass Dounce homogenizer with repeated strokes. The lysates were then clarified by centrifugation at 13,000 rpm for 15 min at 4°C, and the supernatants were transferred to fresh 1.5 ml microcentrifuge tubes for downstream applications. For HA-tag immunoprecipitation, Protein A/G magnetic beads (MCE, Cat# HY-K0201) were pre-washed and incubated with the clarified lysates overnight at 4°C with gentle rotation. Input fractions were collected from the post-incubation supernatants. As a negative control, lysates were incubated overnight at 4°C with anti-IgG antibody (Beyotime, Cat# A7016), followed by incubation with Protein A/G magnetic beads (Beyotime, Cat# P2108) for an additional 10 h at 4°C. After extensive washing, bead-bound proteins were eluted, resuspended in protein loading buffer, denatured at 95°C for 5–10 min, and resolved by SDS-PAGE. Gels were stained with Coomassie Brilliant Blue, and visible protein bands of interest were excised for mass spectrometric analysis.

### siRNA transfection

For siRNA transfection experiments, Lipofectamine RNAiMAX transfection reagent and GibcoTM Opti-MEMTM medium were used. Cultured VSMCs were harvested, resuspended, and mixed with the corresponding siRNA, RNAiMAX transfection reagent, and Opti-MEM medium. After incubation, the mixture was added to cells seeded in a six-well plate for transfection. The transfected cells were subsequently incubated in a cell culture incubator for 24–48 h to assess transfection efficiency and for subsequent experiments. The sequences of the oligo siRNAs used were as follows: si-TPM1-1 (si-TPM1-1 CGGAGAGGUCAGUAACUAA), si-TPM1-2 (CAGACUUUACUGGAGUUAA), and si-TPM1-3 (GGAAAUUCAGGAGAUCCAA).

#### Bioinformatic analyses

Microarray data were analyzed using bioinformatic tools from Assistant for Clinical Bioinformatics (www.aclbi.com). The detailed methods were as follows: The gene expression matrix file, GSE100927_series_matrix.txt.gz, was downloaded from the supplementary section (https://ftp.ncbi.nlm.nih.gov/geo/series/GSE100nnn/GSE100927/suppl/). The microarray data underwent log2 transformation and normalization using the preprocessCore package in R (version 3.4.1). Probes were converted to gene symbols based on the annotation information for the normalized platform data. For genes represented by multiple probes, the average expression value was used. For different datasets or the same dataset across different platforms, only genes with common symbols were extracted. Each datase and platform was marked as a different batch, and batch effects were removed using the removeBatchEffect function of the limma package in R. Adjusted *P* values were calculated to correct for false positive results in the GEO datasets. Differentially expressed RNAs were identified using the criteria ‘Adjusted *P*<0.05 and abs (log(Fold Change)) > 1’. Heatmaps and volcano plots were constructed to visualize differential RNA expression. Gene expression distribution was presented as a boxplot. Statistical difference comparisons between two groups were performed using the Wilcoxon test, while differences among three groups were tested with the Kruskal–Wallis test.

### Statistical analysis

All experiments were repeated at least three times and the values were averaged. Data were expressed as Means ± SEM. Statistical analyses were performed using GraphPad Prism version 9 (GraphPad Software, San Diego, CA, U.S.A.). Differences between two groups were analyzed using an independent two-tailed *t*-test. For comparisons among three or more groups, one-way analysis of variance (ANOVA) was conducted after testing for homogeneity of variances. When ANOVA revealed significant differences, *post hoc* pairwise comparisons were performed using *t*-tests between individual groups. A *P* value of less than 0.05 was considered statistically significant.

## Results

### TSPAN4 expression is up-regulated in human atherosclerotic arteries, ligated mouse carotid arteries with neointimal formation, and PDGF-BB-stimulated VSMCs

**Figure 1:**
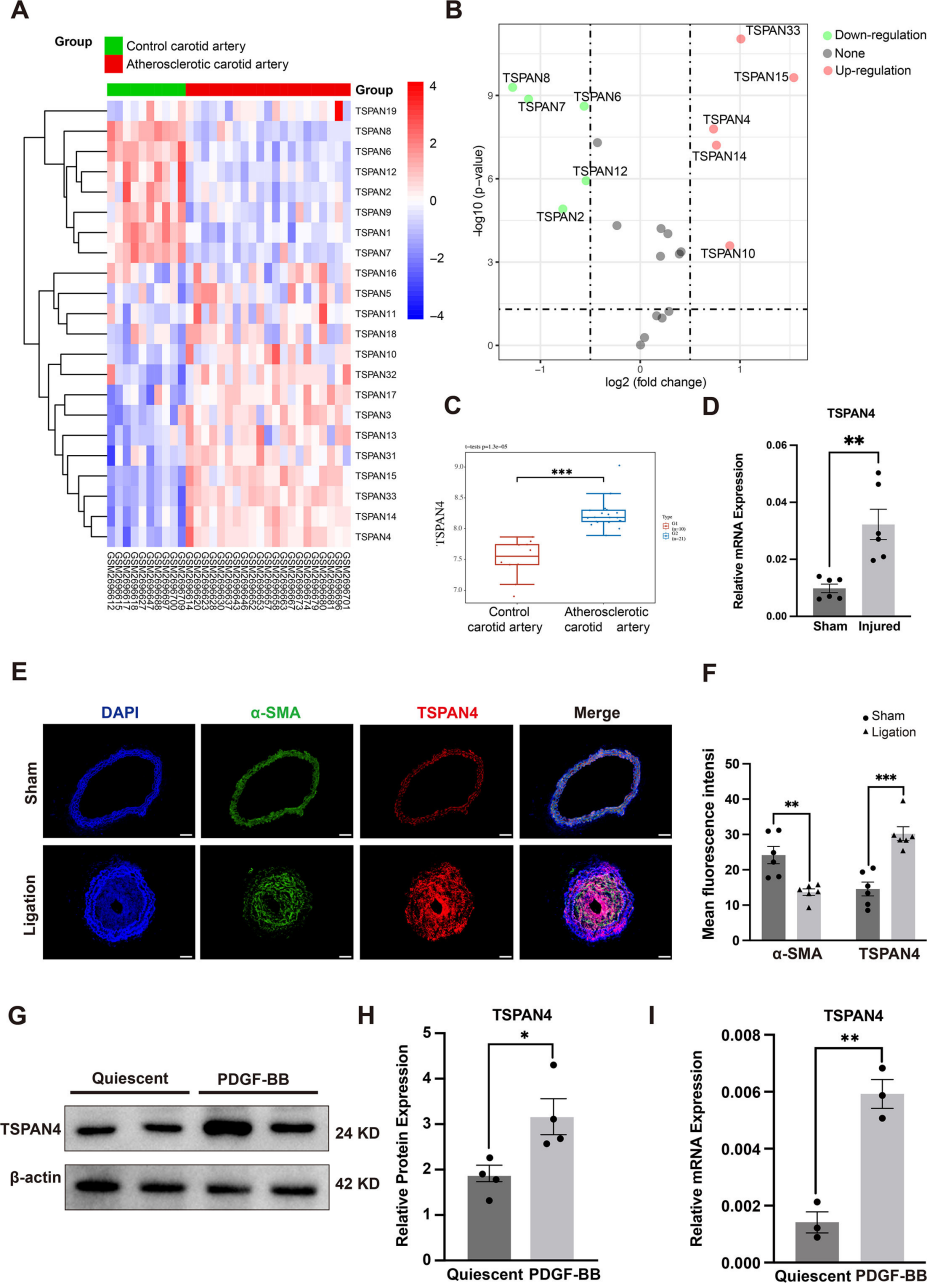
TSPAN4 expression is elevated in human atherosclerotic arteries, ligated mouse carotid arteries with neointimal formation, and PDGF-BB-stimulated vascular smooth muscle cells (VSMCs). **(A**). A heatmap illustrating the expression pattern of TSPAN family genes across different tissue groups (*n* = 10 for the control carotid artery group, *n* = 21 for the atherosclerotic artery group). Colors represent gene expression trends. (**B**). A volcano plot showing the up-regulated and down-regulated TSPAN genes in human atherosclerotic arteries. (**C**). A boxplot of the normalized data showing TSPAN4 gene expression distribution in the different groups.(***P<0.001). (**D**). Relative *Tspan4* mRNA levels in mouse ligated carotid arteries at 14 days post-surgery. one technical replicate measurement per sample was performed for both the sham group (*n* = 6) and the ligation group (*n* = 6). *P<0.05, independent samples *t*-test. (**E, F**). Immunofluorescence staining for TSPAN4 (red) and α-SMA (green) in mouse carotid arteries. Cell nuclei were counterstained with DAPI (blue). Scale bar: 50 μm. Two groups (*n* = 6 per group) were analyzed, **P<0.01, ***P<0.001, independent samples *t*-test. (**G, H**). Cultured VSMCs were serum-starved for 48 h and then stimulated with or not with PDGF-BB (25 ng/ml) for another 24 h. TSPAN4 protein expression levels were detected by western blot. Four independent replicate measurements per sample was performed for both the quiescent group and the PDGF-BB stimulation group (*n* = 2 per group) (*P<0.05), independent samples *t*-test. (**I**). Relative mRNA expression of *TSPAN4* in PDGF-BB-stimulated VSMCs (**P<0.01). All data are shown as means ± SEM. *P*<0.05 was considered statistically significant.

Initially, we obtained microarray data on human atherosclerotic samples (AS) and normal control samples from the GEO database (GSE100927). After processing, we observed that some TSPAN family genes were differentially expressed between normal control and patient samples (Figure 1A), with some showing significant up-regulation or down-regulation (Figure 1B). Notably, *TSPAN4* exhibited the highest expression among the TSPAN family genes, and its levels were markedly elevated in human atherosclerotic arteries (Figure 1C).

Next, we examined the expression levels of TSPAN4 in the ligated carotid arteries of mice. The mRNA expression of TSPAN4 was significantly increased in ligated arteries with neointimal formation compared with that in normal control vessels (Figure 1D). Furthermore, immunofluorescence staining revealed that TSPAN4 expression was significantly higher in ligated arteries than in unligated ones (Figure 1E and F).

Following vascular endothelial injury, activated platelets adhere locally and release PDGF-BB, a classical stimulatory factor. PDGF-BB is widely used *in vitro* to simulate phenotypic switching from a contractile state to a synthetic state. Here, we investigated the effect of PDGF-BB on TSPAN4 expression. Both real-time PCR and western blotting analysis indicated that stimulation with PDGF-BB led to a significantly decreased expression of the contractile phenotype markers smooth muscle α-actin (α-SMA), smooth muscle 22α (SM22α), and CNN1 in VSMCs at both the mRNA and protein levels, indicating that a phenotypic transition from a contractile state to a synthetic one had been induced (Supplementary Figure S1A and Supplementary Figure S1B). Concurrently, the expression of TSPAN4 in PDGF-BB-stimulated VSMCs was increased at both the transcriptional and translational levels (Figure 1G–1I).

### The effects of TSPAN4 on VSMC proliferation, migration, and phenotypic marker expression

**Figure 2:**
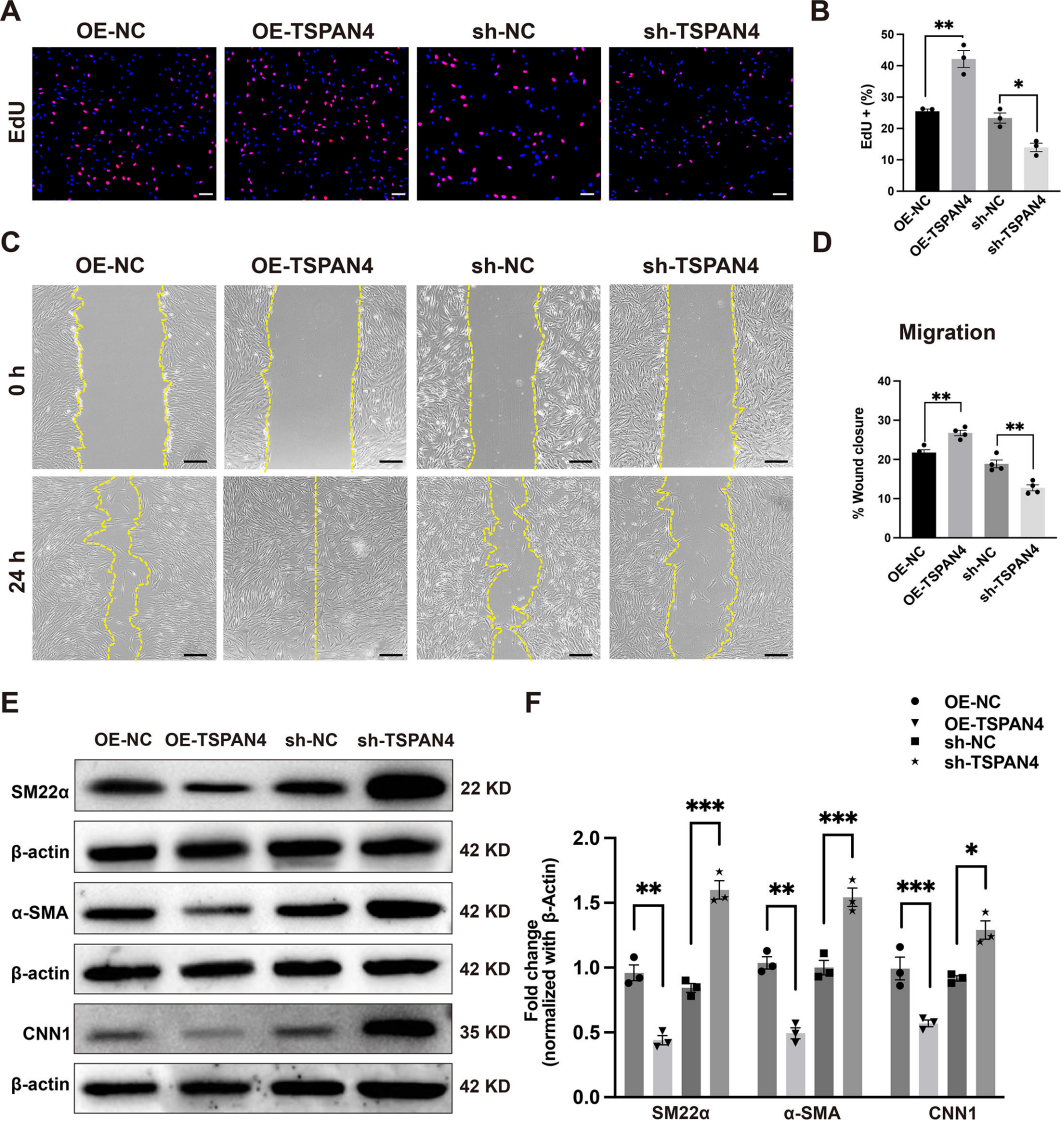
The effects of TSPAN4 on vascular smooth muscle cell (VSMC) proliferation, migration, and phenotypic marker expression. **(A, B**). An EdU assay was conducted to assess the proliferation of VSMCs following TSPAN4 overexpressing or knockdown. EdU staining is shown in red, and cell nuclei are counterstained with DAPI (blue). Scale bar: 100 μm. Four groups (*n* = 3 per group) were analyzed, with each cell sample in each group undergoing three independent replicate measurements. One-way ANOVA analysis was used for overall group comparisons, and an independent samples *t*-test was used to employed for pairwise comparisons (*P<0.05, **P<0.01). (**C, D). A** wound healing assay was undertaken to assess the migratory potential of VSMCs overexpressing TSPAN4 or with TSPAN4 knockdown. The yellow line indicates the cell migration front. Scale bar: 100 μm. Four groups (*n* = 3 per group) were analyzed, with each cell sample in each group undergoing four independent replicate measurements. One-way ANOVA analysis was used for overall group comparisons, and an independent samples *t*-test was used to employed for pairwise comparisons (**P<0.01). (**E, F**). The protein expression levels of contractile marker genes (α-SMA, SM22α, and CNN1) in VSMCs with TSPAN4 overexpression or knockdown were assessed by western blot. Representative immunoblots and densitometric analysis are shown. Four groups (*n* = 3 per group) were analyzed, with each cell sample in each group undergoing three independent replicate measurement. One-way ANOVA analysis was used for overall group comparisons, and an independent samples *t*-test was employed for pairwise comparisons (*P<0.05, **P<0.01, ***P<0.001). All data are shown as means ± SEM. *P*<0.05 was considered statistically significant.

To determine the cellular functions of TSPAN4 in VSMCs *in vitro,* we generated stable lines with either TSPAN4 overexpression or knockdown using lentiviral infection. The efficiency of overexpression or knockdown was confirmed at the protein level, demonstrating successful modulation of TSPAN4 expression in smooth muscle cells (Supplementary Figure S2A and Supplementary Figure S2B). We conducted EdU assays to examine the effect of TSPAN4 on VSMC proliferation. As shown in Figure 2A and B, the knockdown of TSPAN4 inhibited VSMC proliferation, while its overexpression yielded the opposite result. Wound healing assays and Transwell assays were used to assess the influence of TSPAN4 on VSMC migration. The results showed that the knockdown of TSPAN4 suppressed the migratory ability of VSMCs, whereas the opposite trend was observed with TSPAN4 overexpression (Figure 2C and D, Supplementary Figure S2C and Supplementary Figure S2D). We further investigated the expression of markers of the contractile phenotype—α-SMA, SM22α, and CNN1—in both TSPAN4-overexpressing (OE-TSPAN4) and TSPAN4-depleted (sh-TSPAN4) VSMCs. Interestingly, the knockdown of TSPAN4 in VSMCs led to an increase in the expression of these contractile markers, while TSPAN4 overexpression elicited the opposite effect (Figure 2E and F).

#### TSPAN4 knockdown inhibits PDGF-BB-induced phenotypic switching in VSMCs

**Figure 3:**
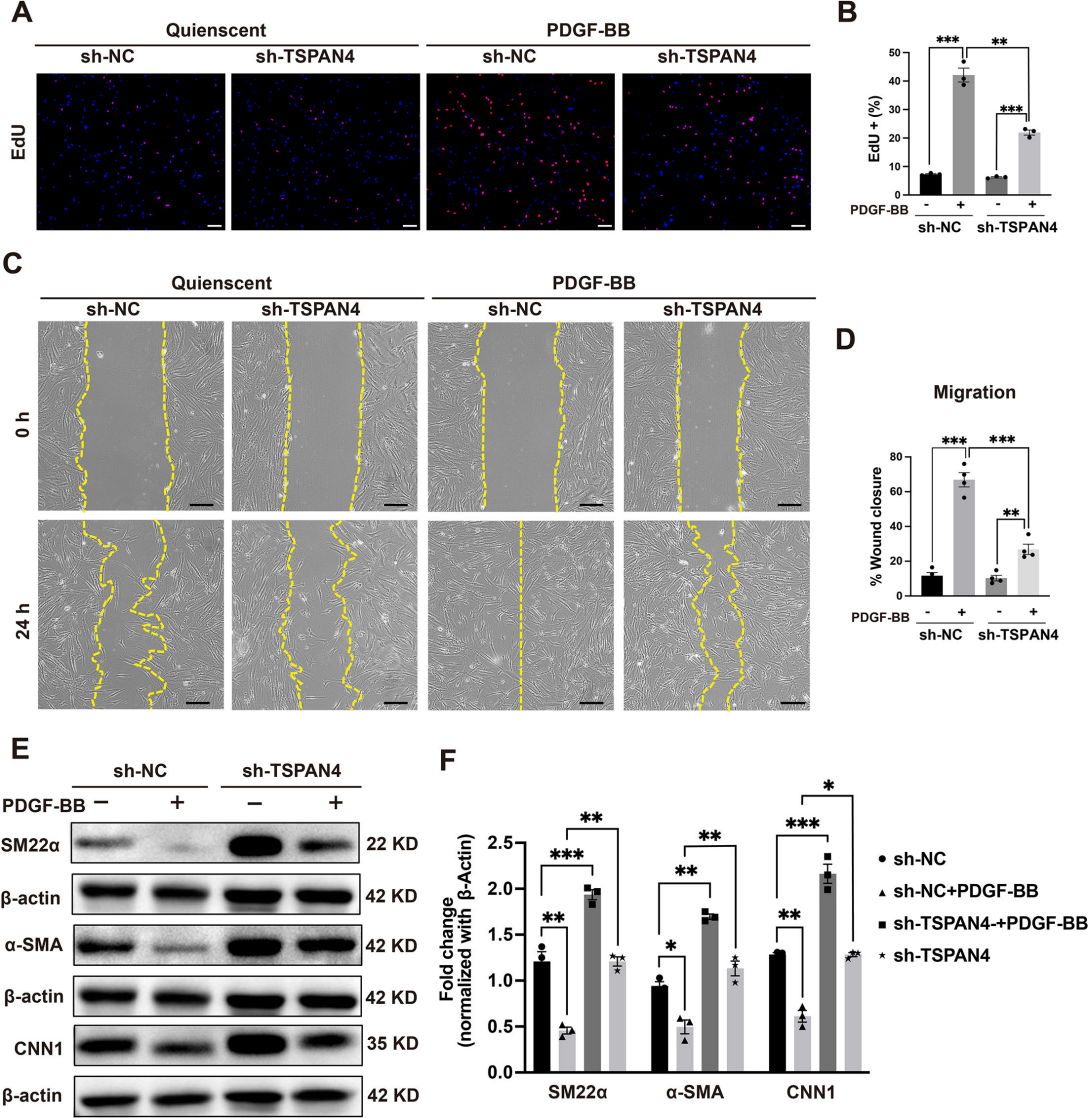
The knockdown of TSPAN4 inhibits PDGF-BB-induced proliferation, migration, and phenotypic switching in vascular smooth muscle cells (VSMCs). Cultured VSMCs were serum-starved for 48 hours and then stimulated or not with PDGF-BB (25 ng/mL) for 24 h. (**A, B**). An EdU assay was conducted to assess the PDGF-BB-induced proliferation of VSMCs with or without TSPAN4 knockdown. EdU staining is shown in red, and cell nuclei are counterstained with DAPI (blue). Scale bar: 100 μm. Four groups (*n* = 3 per group) were analyzed, with each cell sample in each group undergoing three independent replicate measurements. One-way ANOVA analysis was used for overall group comparisons, and an independent samples *t*-test was employed for pairwise comparisions (**P<0.01, ***P<0.001). (**C, D**). A wound healing assay was performed to assess the effect of PDGF-BB on migration in VSMCs with or without TSPAN4 knockdown. The yellow line represents the cell migration front. Scale bar: 100 μm. Four groups (*n* = 3 per group) were analyzed, with each cell sample in each group undergoing four independent replicate measurements. One-way ANOVA analysis was used for overall group comparisons, and an independent samples *t*-test was employed for pairwise comparisions (**P<0.01, ***P<0.001). (**E, F**). The expression of contractile phenotype markers (α-SMA, sm22α, and CNN1) in PDGF-BB-treated VSMCs with or without TSPAN4 knockdown was assessed by western blot. Representative immunoblots and densitometric analysis are presented. Four groups (*n* = 3 per group) were analyzed, with each cell sample in each group subjected to three independent replicate measurements. One-way ANOVA analysis was used for overall group comparisons, and an independent samples *t*-test was employed for pairwise comparisons (*P<0.05, **P<0.01, ***P<0.001). All data are shown as means ± SEM. *P*<0.05 was considered statistically significant.

To further elucidate the role of TSPAN4 in PDGF-BB-induced phenotypic switching in VSMCs, we treated both sh-NC (negative control) and sh-TSPAN4 VSMCs with PDGF-BB. As expected, PDGF-BB treatment promoted proliferation and migration in control cells (sh-NC), consistent with the induction of a synthetic phenotype. However, these effects were significantly attenuated in TSPAN4-deficient VSMCs (sh-TSPAN4) (Figure 3A–3D).

At the molecular level, PDGF-BB treatment led to a marked reduction in the expression of markers of the contractile phenotype in sh-NC cells. Notably, this PDGF-induced down-regulation was significantly mitigated in TSPAN4-knockdown cells (Figure 3E and F). These results suggested that a decline in TSPAN4 levels partially counteracts PDGF-BB-induced phenotypic switching, highlighting a modulatory role for TSPAN4 in this process.

#### TSPAN4 influences the cytoskeletal organization in VSMCs

**Figure 4:**
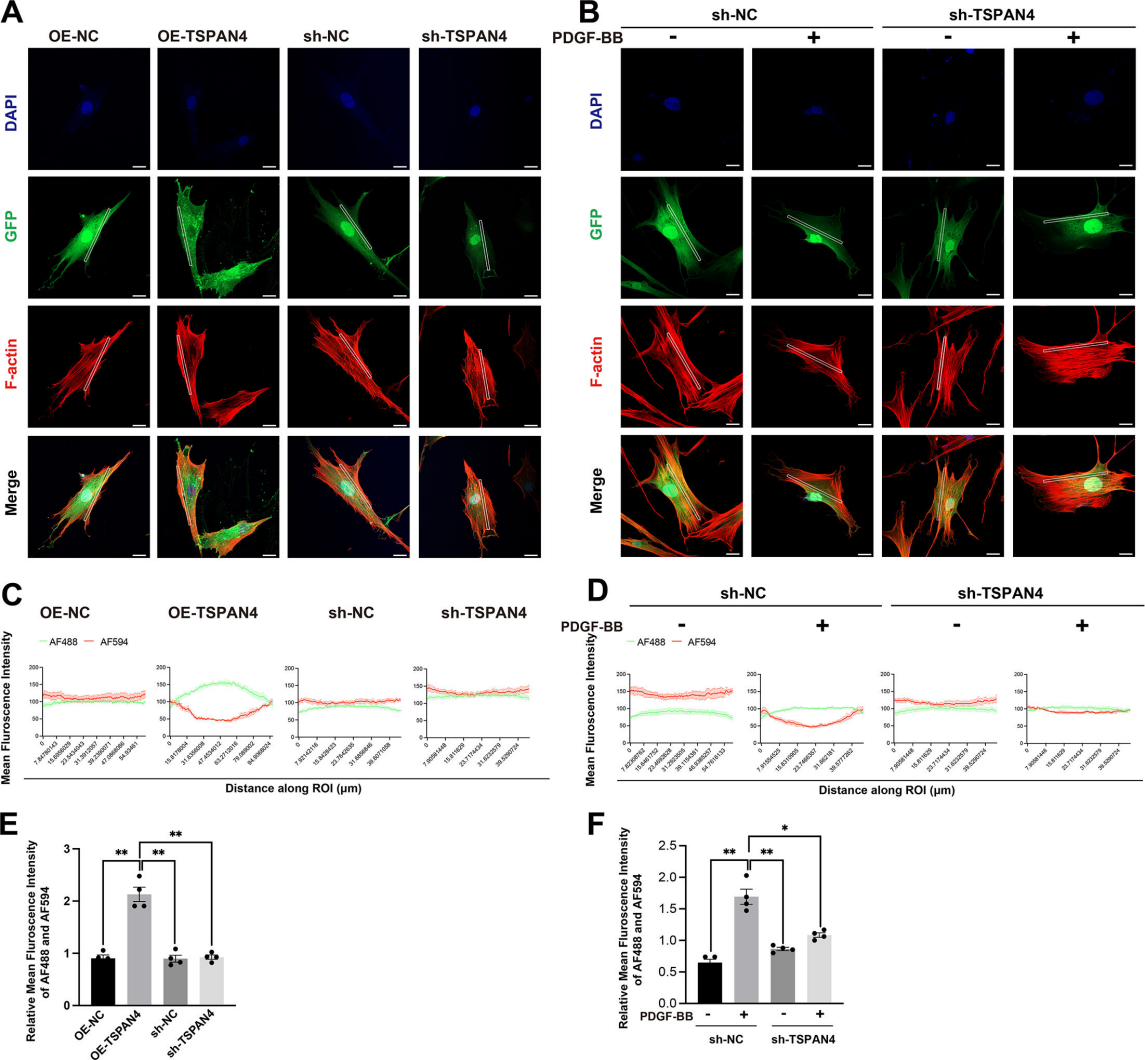
The influence of TSPAN4 on cytoskeletal organization in vascular smooth muscle cells (VSMCs). **(A**). Immunofluorescence analysis of cytoskeletal organization in TSPAN4-overexpresing and TSPAN4-knockdown VSMCs. In the TSPAN4 overexpression vector, TSPAN4 is expressed as a TSPAN4-GFP fusion protein. GFP (green) represents GFP (for the OE-NC/sh-NC/sh-TSPAN4 vectors) or TSPAN4 (for the OE-TSPAN4 vector), F-actin (red) marks the cytoskeleton, and cell nuclei are counterstained with DAPI (blue). Representative images are shown. Scale bar: 20 μm. (**B**). Cultured VSMCs were serum-starved for 48 h and then stimulated or not with PDGF-BB (25 ng/ml) for 30 minutes. Immunofluorescence staining was used to assess cytoskeletal organization in TSPAN4-knockdown VSMCs treated or not with PDGF-BB. GFP (green) represents GFP (for the OE-NC/sh-NC/sh-TSPAN4 vectors) or TSPAN4 (for the OE-TSPAN4 vector), F-actin (red) marks the cytoskeleton, and cell nuclei are counterstained with DAPI (blue). Representative images are shown. (**C, D**) Profile plots showing the mean fluorescence intensity of TSPAN4 (green) and F-actin (red) along rectangular regions of interest (ROIs). (**E, F**) AF488/AF594 intensity ratios computed from ROIs across different groups. Scale bar: 20 μm. One-way ANOVA was used for overall group comparisons, and an independent samples *t*-test was employed for pairwise comparisons. Data represent means ± SEM. *P<0.05, ** *P*<0.01.

Given the observed differences in cell morphology between contractile and synthetic VSMCs (Supplementary Figure S2), we next stained the cytoskeleton using Actin-Tracker Red-Rhodamine. Notably, TSPAN4 overexpression led to a reduction in the number of stress fibers, and these exhibited a more sparse distribution (Figure 4A). Additionally, we noted that TSPAN4 primarily localized to the cytoplasm and cell membrane. Analysis of merged images revealed a strong negative correlation between elevated TSPAN4 expression and reduced F-actin staining. Conversely, TSPAN4 knockdown resulted in an increase in F-actin density (Figure 4A).

Given the critical role of cytoskeletal organization during phenotypic transitions, understanding these processes significantly contributes to our knowledge about the physiological functions and pathological changes of VSMCs. To further explore the role of TSPAN4 in cytoskeletal dynamics during VSMC phenotypic transitions, we treated both control and TSPAN4-knockdown VSMCs with PDGF-BB. No differences in F-actin staining were observed between control and PDGF-BB-stimulated VSMCs following TSPAN4 knockdown, suggestive of a stable cytoskeletal arrangement (Figure 4B).

To quantitatively examine this spatial relationship between TSPAN4 expression and cytoskeletal arrangement, we performed an image-based spatial fluorescence analysis using intensity profile curves along the ROI to visualize spatial co-distribution or inverse-distribution patterns. Statistical evaluation revealed that F-actin intensity was significantly reduced in OE-TSPAN4 cells compared with that in control cells, whereas the TSPAN4/F-actin ratio was significantly increased (Figure 4E), reflecting an inverse spatial relationship between TSPAN4 expression and F-actin abundance. Intensity profile curve analysis further supported these findings, showing that regions with high TSPAN4 intensity coincided with troughs in the F-actin signal (Figure 4C). Similar patterns of F-actin disruption patterns were observed in sh-NC cells treated with PDGF-BB, consistent with PDGF’s established role in actin disassembly (Figure 4B, D and F).

#### TSPAN4 controls VSMC phenotypic switching and cytoskeletal organization by targeting TPM1

**Figure 5:**
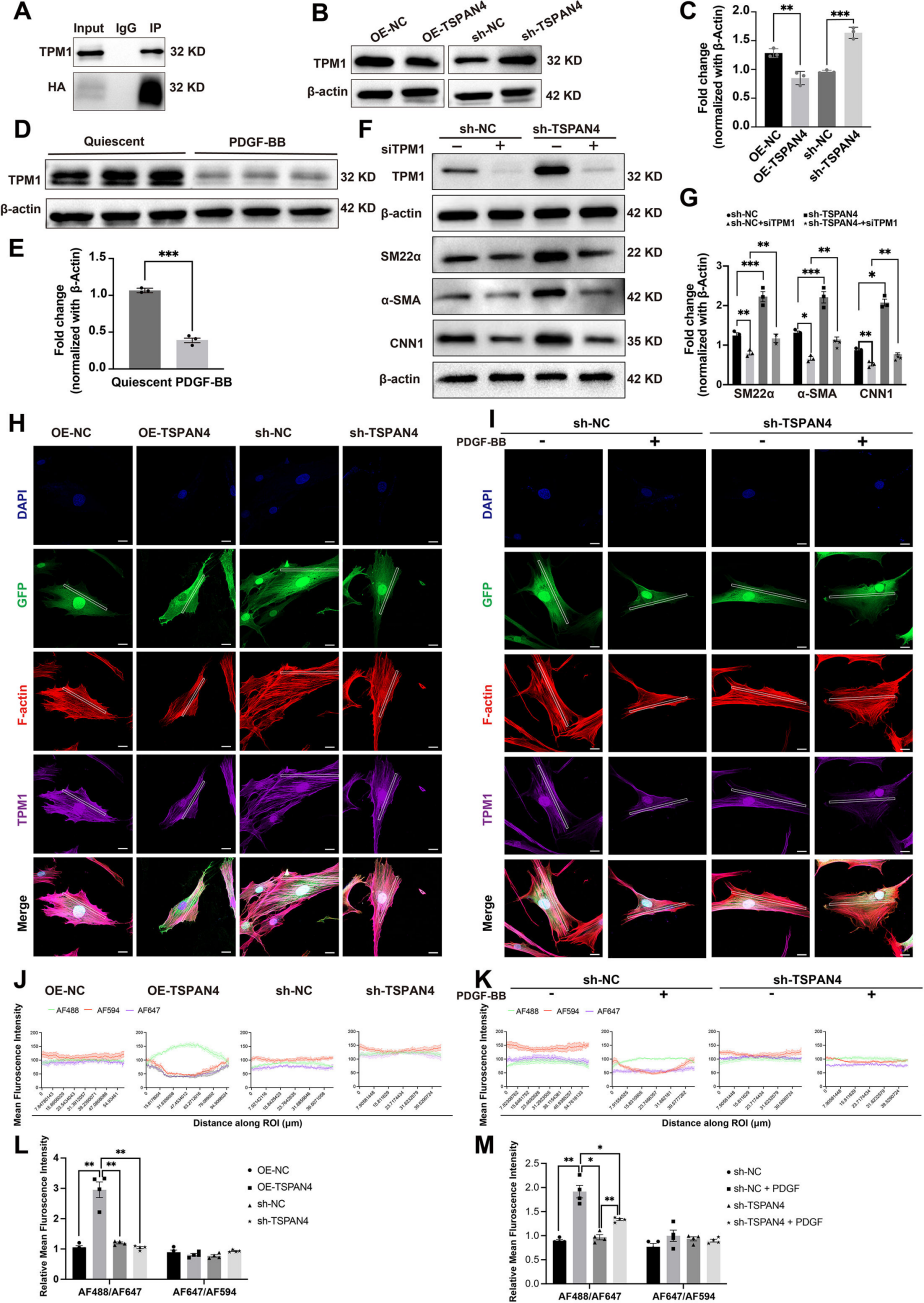
TSPAN4 regulates phenotypic switching in vascular smooth muscle cells by targeting TPM1. **(A**). Western blot was used to validate the pull-down of TPM1. (**B, C**). The protein expression levels of TPM1 in TSPAN4-overexpressing and TSPAN4-knockdown VSMCs. Representative immunoblots and densitometric analysis are presented. Four groups (*n* = 3 per group) were analyzed, with each cell sample in each group undergoing three independent replicate measurements. One-way ANOVA was used for overall group comparisons, and an independent samples *t*-test was employed for pairwise comparisons (**P<0.01, ***P<0.001). (**D, E**). The protein expression levels of TPM1 in VSMCs stimulated with PDGF-BB. Representative immunoblots and densitometric analysis are shown. The quiescent group (*n* = 3) and the PDGF-BB group (*n* = 3) each underwent three independent replicate measurements per cell sample. ***P<0.001, independent samples *t*-test. (**F, G**). TPM1 was silenced in TSPAN4-knockdown VSMCs using its siRNA, and the protein levels of TPM1 and contractile phenotype marker genes were assessed by western blots. Representative immunoblots and densitometric analysis are presented. Four groups (*n* = 3 per group) were analyzed, with each cell sample in each group being subjected to three independent replicate measurements. *P<0.05, **P<0.01, ***P<0.001, independent samples *t*-test. (**H**) Immunofluorescence staining was used to detect the cytoskeletal organization and TPM1 in TSPAN4-overexpressing or TSPAN4-knockdown VSMCs. GFP (green) represents GFP (for the OE-NC/sh-NC/sh-TSPAN4 vectors) or TSPAN4 (for the OE-TSPAN4 vector), F-actin (red) marks the cytoskeleton, TPM1 is shown in purple, and cell nuclei are counterstained with DAPI (blue). Scale bar: 20 μm. (**I**). Cultured VSMCs were serum-starved for 48 h and then stimulated or not with PDGF-BB (25 ng/mL). Immunofluorescence staining was used to detect cytoskeletal organization and TPM1 in TSPAN4-knockdown VSMCs treated or not with PDGF-BB. GFP (green) represents GFP (for the OE-NC/sh-NC/sh-TSPAN4 vectors) or TSPAN4 (for the OE-TSPAN4 vector), F-actin (red) marks the cytoskeleton, TPM1 is stained purple, and cell nuclei are counterstained with DAPI (blue). (**J, K**) Profile plots showing the mean fluorescence intensity of TSPAN4 (green), F-actin (red), and TPM1 (purple) along rectangular regions of interest (ROIs). (**L, M**) AF488/AF594 and AF647/AF594 intensity ratios computed from ROIs across different groups. Data represent means ± SEM. *P<0.05, ** *P*<0.01. Scale bar: 20 μm. One-way ANOVA was used for overall group comparisons, and an independent samples *t*-test was employed for pairwise comparisons. *P*<0.05 was considered statistically significant.

To elucidate the molecular mechanism underlying how TSPAN4 modulates cytoskeletal changes and phenotypic switching in VSMCs, we introduced an HA Flag (HA) tag to the TSPAN4 overexpression vector. IP was then performed using HA-tagged beads to pull down TSPAN4 and its binding proteins, with IgG antibody added as a control. The precipitated proteins were separated by SDS-PAGE, stained with Coomassie Brilliant Blue, and subjected to mass spectrometric analysis to identify the TSPAN4-interacting proteins (Supplementary Figure 3A). The results revealed that multiple members of the tropomyosin (TPM) family interact directly with TSPAN4, with TPM1 and TPM3 exhibiting the highest number of paired peptides. To confirm that TSPAN4 directly interacts with TPM1 and TPM3, we subsequently performed co-IP experiments. We found that TPM1 was successfully pulled down in the IP group, demonstrating that it directly interacted with TSPAN4 (Figure 5A). However, no direct interaction was detected between TSPAN4 and TPM3 (Supplementary Figure S3B).

Further experiments revealed that the overexpression of TSPAN4 in VSMCs led to a decrease in TPM1 expression, whereas its knockdown exerted the opposite effect (Figure 5B and C). These results indicated that TSPAN4 regulates the expression of TPM1, and that TPM1 may play an important role in the TSPAN4-mediated VSMC phenotypic switching. Furthermore, the expression of TPM1 was decreased in VSMCs following PDGF-BB treatment (Figure 5D and E), indicating its potential role in PDGF-BB-induced VSMC phenotypic switching.

To validate the impact of the TSPAN4/TPM1 interaction on phenotypic switching, we knocked down TPM1 in VSMCs (Supplementary Figure S3C) and measured the changes in the protein levels of contractile phenotype markers. As expected, the knockdown of TPM1 in the control (sh-NC) group resulted in a slight decrease in the expression of these markers. However, the knockdown of TPM1 in sh-TSPAN4 VSMCs markedly attenuated the TSPAN4 knockdown (sh-TSPAN4)-induced increase in contractile phenotype marker expression (Figure 5F and G). Consistently, in VSMCs overexpressing TSPAN4, TPM1 knockdown further decreased the levels of contractile phenotype markers (Supplementary Figure S3D and Supplementary Figure S3E). Taken together, these results demonstrate that TPM1 is indispensable for maintaining the contractile phenotype of VSMCs and that its down-regulation exacerbates TSPAN4-driven phenotypic switching.

It has been reported that TPM1 regulates the structure and function of cytoskeletal actin filaments and inhibits VSMC proliferation and migration [33]. Given that we found that TSPAN4 induced cytoskeletal reorganization in VSMCs, we wondered whether these effects of TSPAN4 could be mediated through TPM1. To test this possibility, we performed immunofluorescence staining to investigate the localization of TPM1 and stress fibers in VSMCs. Interestingly, we found that TPM1 colocalized with stress fibers, and that the expression of TPM1 displayed a negative correlation with that of TSPAN4 (Figure 5H). We further treated sh-TSPAN4 VSMCs with PDGF-BB and performed immunofluorescence staining for TPM1 and F-actin. In the control (sh-NC) group, TPM1 expression was decreased with PDGF-BB treatment, accompanied by a corresponding decrease in F-actin staining. In contrast, TPM1 expression was increased in sh-TSPAN4 VSMCs, alongside an increase in F-actin staining and a tighter F-actin filament arrangement (Figure 5I). Quantitative analysis showed that the TSPAN4/TPM1 ratio (AF488/AF647) was elevated in OE-TSPAN4 VSMCs and sh-NC VSMCs treated with PDGF-BB (Figure 5J and K), with profile curve analysis again revealing inverse spatial distributions between TSPAN4 and TPM1 (Figure 5L and M).

These data collectively support that TSPAN4 negatively regulates stress fiber integrity and TPM1 expression and may play a role in cytoskeletal remodeling during VSMC phenotypic switching.

#### TSPAN4 deficiency attenuates the neointima formation in ligated mouse carotid arteries

**Figure 6:**
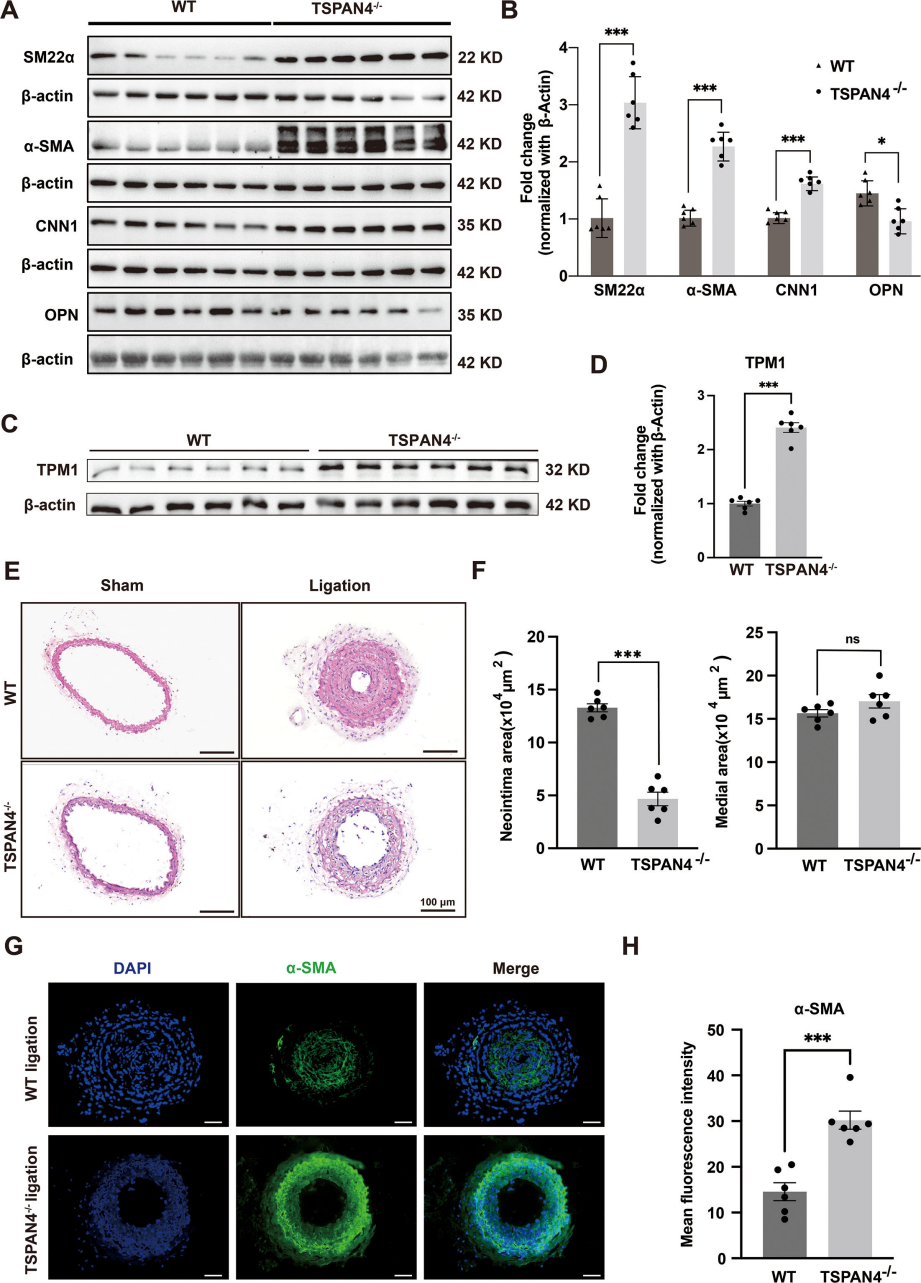
TSPAN4 deficiency attenuates neointima formation in ligated mouse carotid arteries. **(A, B**). Arterial tissues from wildtype (WT) and *Tspan4* ^−/−^ mice were extracted, and the protein levels of contractile phenotype marker genes (α-SMA, SM22α, and CNN1) and the synthetic phenotype marker gene OPN were detected by western blot. Representative immunoblots and densitometric analysis are presented. Two groups (*n* = 6 per group) were analyzed, with each undergoing one technical replicate measurement per sample. *P<0.05, **P<0.01, ***P<0.001, independent samples *t*-test. (**C, D**). The protein expression of TPM1 in arterial tissues of WT and *Tspan4* ^−/−^ mice was detected by western blot. Representative immunoblots and densitometric analysis are presented. Two groups (*n* = 6 per group) were analyzed, with each undergoing one technical replicate measurement per sample. ***P<0.001, independent samples *t*-test. (**E, F**). Hematoxylin and eosin (H&E) staining of carotid artery tissues from WT and *Tspan4* ^−/−^ mice, along with the statistical analysis of neointima and media areas. The neointima area and the ratio of intima to media were quantified. Two groups (*n* = 6 per group) were analyzed, with each undergoing one technical replicate measurement per sample. ***P<0.001, ns: not statistically significant; independent samples *t*-test. (**G, H**). Immunofluorescence staining of carotid artery tissues from WT and *Tspan4* ^−/−^ mice after carotid artery ligation, along with fluorescence intensity analysis of α-SMA expression (green). Cell nuclei were counterstained with DAPI (blue). Scale bar: 50 μm. Two groups (*n* = 6 per group) were analyzed, with each animal sample in each group undergoing three technical replicate measurements. ***P<0.001, independent samples *t*-test. All data are shown as means ± SEM. *P*<0.05 was considered statistically significant.

To validate the function of TSPAN4 *in vivo*, we employed a globally TSPAN4 knockout mouse model to investigate VSMC phenotypic switching and neointima formation (Supplementary Figure S4A and Supplementary Figure S4B). In the vascular tissues of *Tspan4* ^−/−^ mice, the expression of contractile phenotype markers (α-SMA, SM22α, and CNN1) was significantly up-regulated, while that of the synthetic phenotype markers OPN was down-regulated. These findings indicated that TSPAN4 deficiency promotes a differentiated, contractile phenotype in VSMCs in mice (Figure 6A and B). TPM1 expression was also up-regulated in *Tspan4* ^−/−^ mice (Figure 6C and D), further supporting that TPM1 is involved in TSPAN4-regulated VSMCs phenotypic switching *in vivo*.

We then established a vascular injury model by ligating the carotid arteries of WT and *Tspan4* ^−/−^ mice and monitored neointima formation in the vasculature of the animals. The artificial obstruction of blood flow in mouse carotid arteries led to augmented endothelial cell damage and permeability, accompanied by increased secretion of inflammatory substances and growth factors. This, in turn, prompted phenotypic switching in VSMCs, followed by their migration from the media to the intima, proliferation, and deposition of extracellular matrix, resulting in intimal hyperplasia. H&E staining of carotid artery sections at 21 days post-ligation revealed that neointima formation was significantly reduced in *Tspan4* ^−/−^ mice compared with that in their WT counterparts. The carotid arteries of the non-ligation group remained smooth and intact and exhibited a more regular arrangement of smooth muscle cells in the media (Figure 6E and F). Immunofluorescence results staining indicated that neointimal hyperplasia was reduced while the expression of α-SMA was increased in *Tspan4* ^−/−^ mice post-carotid artery ligation (Figure 6G and H).

## Discussion

**Figure 7:**
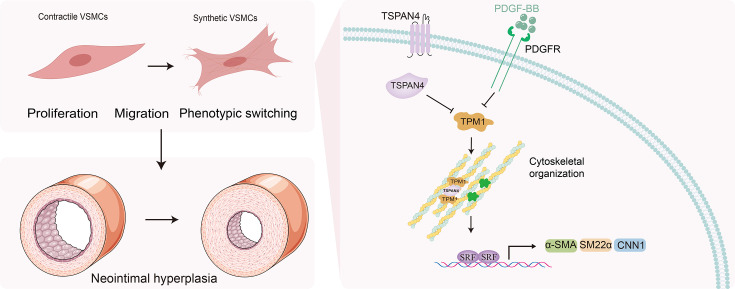
Graphical summary: TSPAN4 controls vascular smooth muscle cell (VSMC) phenotypic switching and intimal hyperplasia by targeting tropomyosin-1 (TPM1)-mediated cytoskeletal organization. The expression of TSPAN4 is elevated in PDGF-BB-stimulated VSMCs or VSMCs in injured vessels, leading to the suppression of TPM1 and a consequent reduction in cytoskeletal stability. This destabilization inhibits the expression of contractile phenotype marker genes in VSMCs via cytoskeletal reorganization-modulated SRF-dependent transcription, ultimately leading to synthetic phenotypic switching in VSMCs. The switched cells display enhanced proliferation and migration, contributing to vascular neointima formation and the development of vascular disease.

The major findings of the present study revealed that TSPAN4 expression is elevated in atherosclerotic arteries and in ligated mouse carotid arteries with neointimal formation. The knockdown or deficiency of TSPAN4 inhibited VSMC proliferation, migration, and phenotypic switching, ultimately leading to reduced neointima formation. The key underlying mechanism was found to involve TPM1-mediated cytoskeletal organization. Importantly, this study represents the first report implicating TSPAN4, a protein whose expression is elevated in diseased human and mouse arteries, as a novel regulator of neointima formation during vascular remodeling and disease (Figure 7).

While TSPAN4 has previously been confirmed to play roles in diverse biological processes, including cancer development [25] and the formation of migrasome formation [27,34,35], whether it also contributes to the development of cardiovascular diseases remains unknown. In this study, using bioinformatics analysis, we found that TSPAN4 expression is up-regulated in diseased human arteries. Our results further revealed that TSPAN4 expression is also up-regulated in ligated mouse carotid arteries *in vivo* and in PDGF-BB-stimulated VSMCs *in vitro*. These findings suggest that TSPAN4 may play a role in vascular diseases.

In this work, we observed that TSPAN4 overexpression promoted VSMC proliferation, migration, and synthetic phenotype switching, while TSPAN4 knockdown inhibited these processes. Synthetic phenotype switching in VSMCs, followed by enhanced VSMC proliferation and migration, is a critical cellular event in the development of many vascular diseases. *In vivo* experiments showed that the knockout of TSPAN4 led to increased expression of contractile phenotype markers, suggesting that TSPAN4 functions as an inducer of vascular disease. Notably, TSPAN4 knockout did not affect vascular development (Figure 6). Combined, these properties make TSPAN4 a potential therapeutic target for the prevention and treatment of vascular diseases associated with VSMC proliferation, migration, and phenotypic switching, such as atherosclerosis, hypertension, restenosis after angioplasty, and diabetic vascular complications [36].

PDGF-BB plays a pivotal role in stimulating VSMC proliferation, migration, and phenotypic switching. Our study revealed that stimulation with PDGF up-regulated the expression of TSPAN4, consistent with its role in promoting the above processes, whereas TSPAN4 knockdown exerted the opposite effect.

While TSPAN4 is generally considered a membrane protein because it possesses four transmembrane domains, it can also be localized in the cytoplasm and possibly within organelles. Notably, many tetraspanin family proteins interact with other membrane proteins, such as integrins [21], which regulate VSMC phenotypic switching through their effects on the extracellular matrix and cell migration [37]. However, our preliminary results did not identify an association between TSPAN4 and integrins, suggesting that TSPAN4 may not act through membrane-targeted integrins. Further investigations are warranted to unravel the precise mechanisms by which TSPAN4 modulates VSMC phenotypic transitions.

Using GFP-tagged constructs, we observed that TSPAN4 is distributed in both the cell membrane and cytoplasm (Figure 5). Our analysis showed that areas with high TSPAN4 expression exhibited cytoskeletal changes characterized by a loose stress fiber arrangement, a key feature in PDGF-BB-induced VSMC phenotypic switching [11–13]. Stress fibers primarily consist of actin filaments and play essential roles in cytoskeletal organization. Synthetic VSMCs contain fewer, shorter, and/or weaker stress fibers than contractile VSMCs, indicative of reduced mechanical support and cellular stability. During a phenotypic transition toward a contractile phenotype, stress fibers tend to increase in number, length, and stability, which enhances the contractile ability and overall mechanical properties of VSMCs [11,13,14]. Moreover, the expression of contractile phenotype marker genes (αSMA and SM22α) is regulated *via* cytoskeletal reorganization-modulated, serum response factor (SRF)-dependent transcription [38,39]. Cytoskeletal dynamics can also modulate VSMC proliferation, migration, phenotypic switching, and vascular remodeling [10,40]. In our study, the knockout of TSPAN4 led to the up-regulation of contractile phenotype marker genes expression without significantly affecting the cytoskeletal arrangement in VSMCs. Furthermore, TSPAN4 knockout inhibited the PDGF-BB-induced down-regulation of contractile phenotype marker genes and cytoskeletal rearrangements. These findings suggest that TSPAN4 regulates VSMC phenotypic switching through its impact on cytoskeletal organization.

Although tetraspanin proteins primarily engage in cytoskeletal interactions at the cell membrane, our study suggests that TSPAN4 may inhibit cytoskeletal polymerization through an alternative mechanism. To understand how TSPAN4 influences cytoskeletal organization, we undertook IP and mass spectrometric analyses and identified TPM1, a protein involved in muscle contraction and cytoskeletal structure, as a TSPAN4-interacting partner. TPM1 regulates biological structure and function by modulating muscle contraction and cytoskeleton formation and maintenance, thereby exerting essential functions such as structural support and signal transduction [41,42]. Studies have reported that TPM1 regulates the dynamics and function of cytoskeletal actin filaments and inhibits VSMC proliferation and migration, suggesting that it exerts a protective effect in arteriosclerosis [33]. We found that the overexpression of TSPAN4 suppressed TPM1 expression, whereas its knockdown resulted in increased TPM1 levels. Furthermore, TPM1 expression was also observed to be elevated in vascular tissues from TSPAN4 knockout mice, supporting that TSPAN4 is involved in the regulation of TPM1 expression. Immunofluorescence assays also showed that TPM1 co-localized with stress fibers. Moreover, the knockdown of TPM1 using siRNA reversed the TSPAN4 knockdown-mediated increase in the expression of phenotype contractile markers. Collectively, these findings demonstrated that TSPAN4 knockdown blocks VSMC phenotypic switching by stabilizing the cytoskeleton through its regulatory effect on TPM1.

Interestingly, although it interacted with TSPAN4, the expression of TPM1 was down-regulated in TSPAN4-overexpressing VSMCs. The precise mechanism underlying this effect remains unclear and warrants further investigation.

In summary, our study was the first to demonstrate that TSPAN4 plays a crucial role in VSMC phenotypic switching through TPM1-mediated cytoskeletal organization. The loss of TSPAN4 maintains the differentiated phenotype of VSMCs and suppresses neointima formation, identifying TSPAN4 as a potential therapeutic target for preventing intimal hyperplasia and a range of vascular diseases.

**Clinical perspectives**

- Vascular smooth muscle cell (VSMC) phenotypic switching is a pivotal driver of intimal hyperplasia in cardiovascular diseases; however, the role of tetraspanin family proteins, such as TSPAN4, in this process remains unexplored and warrants mechanistic investigations.
- TSPAN4 expression is elevated in human atherosclerotic lesions and injury-induced neointimal hyperplasia, promoting VSMC proliferation and migration by disrupting TPM1-mediated cytoskeletal organization. In contrast, its depletion attenuates pathological vascular remodeling.
- Targeting TSPAN4 may represent a novel therapeutic strategy for suppressing VSMC-driven vascular remodeling, potentially mitigating clinical conditions such as post-angioplasty restenosis and the progression of atherosclerosis.

## Supplementary material

Online supplementary figure 1

Online supplementary figure 2

Online supplementary figure 3

Online supplementary figure 4

Online supplementary table 1

## Data Availability

The datasets used and/or analysed during the current study are available from the corresponding author on reasonable request.

## Open Access

This article has been published open access under our Subscribe to Open programme, made possible through the support of our subscribing institutions, learn more here: https://portlandpress.com/pages/open_access_options_and_prices#conditional

## Abbreviations

CNN1: Calponin 1
OPN: Osteopontin
PDGF-BB: Platelet-derived growth factor subunit B homodimer
SM22α: Smooth muscle 22α
SRF: Serum response factor
TPM1: Tropomyosin-1
TSPAN4: Tetraspanin 4
VSMCs: Vascular smooth muscle cells
VSMCs: Human aortic smooth muscle cells
WT: Wildtype
α-SMA: Smooth muscle α-actin
