# Supplementary figures and images for: TSPAN4 controls vascular smooth muscle cell phenotypic switching and intimal hyperplasia by targeting TPM1-regulated cytoskeletal organization
Source: Clin Sci (Lond). 2025 Oct 8;139(19):1145–61. doi: 10.1042/CS20255833 (PMC12687436; doi:10.1042/CS20255833)

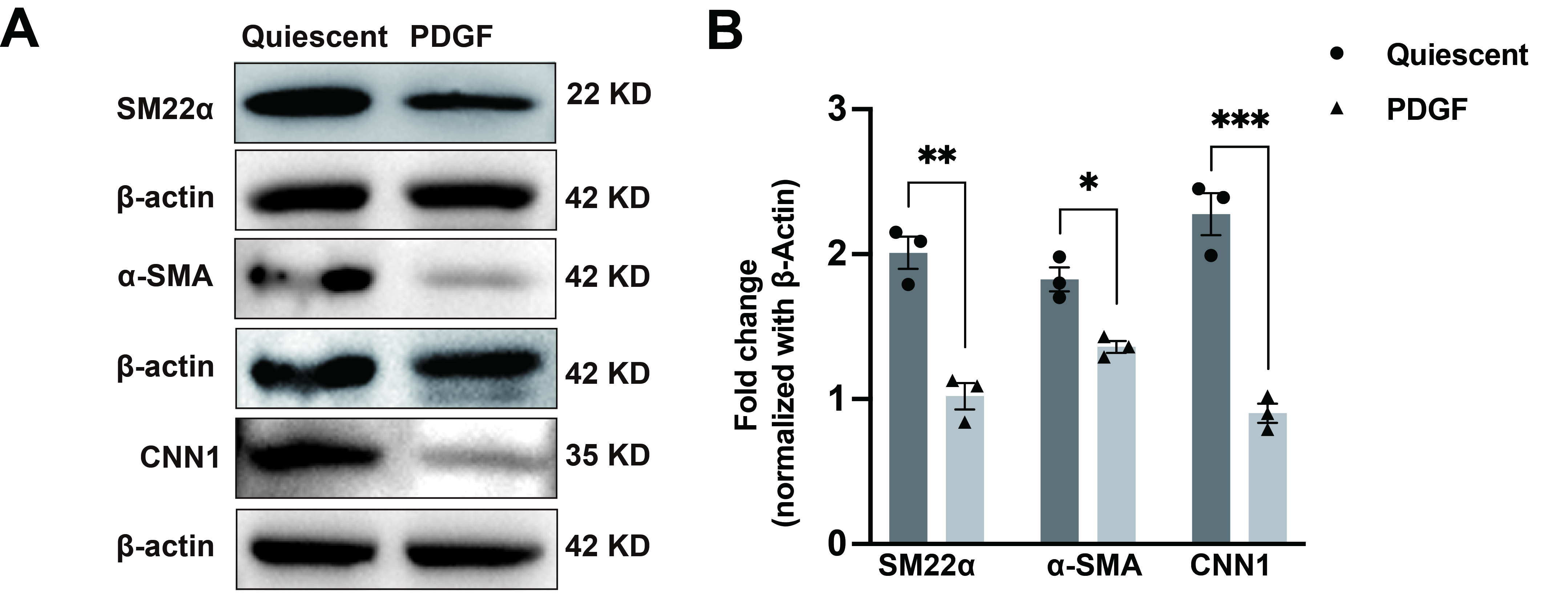

Supplement: Online supplementary figure 1 [file cs-139-19-CS20255833-s001.jpg]

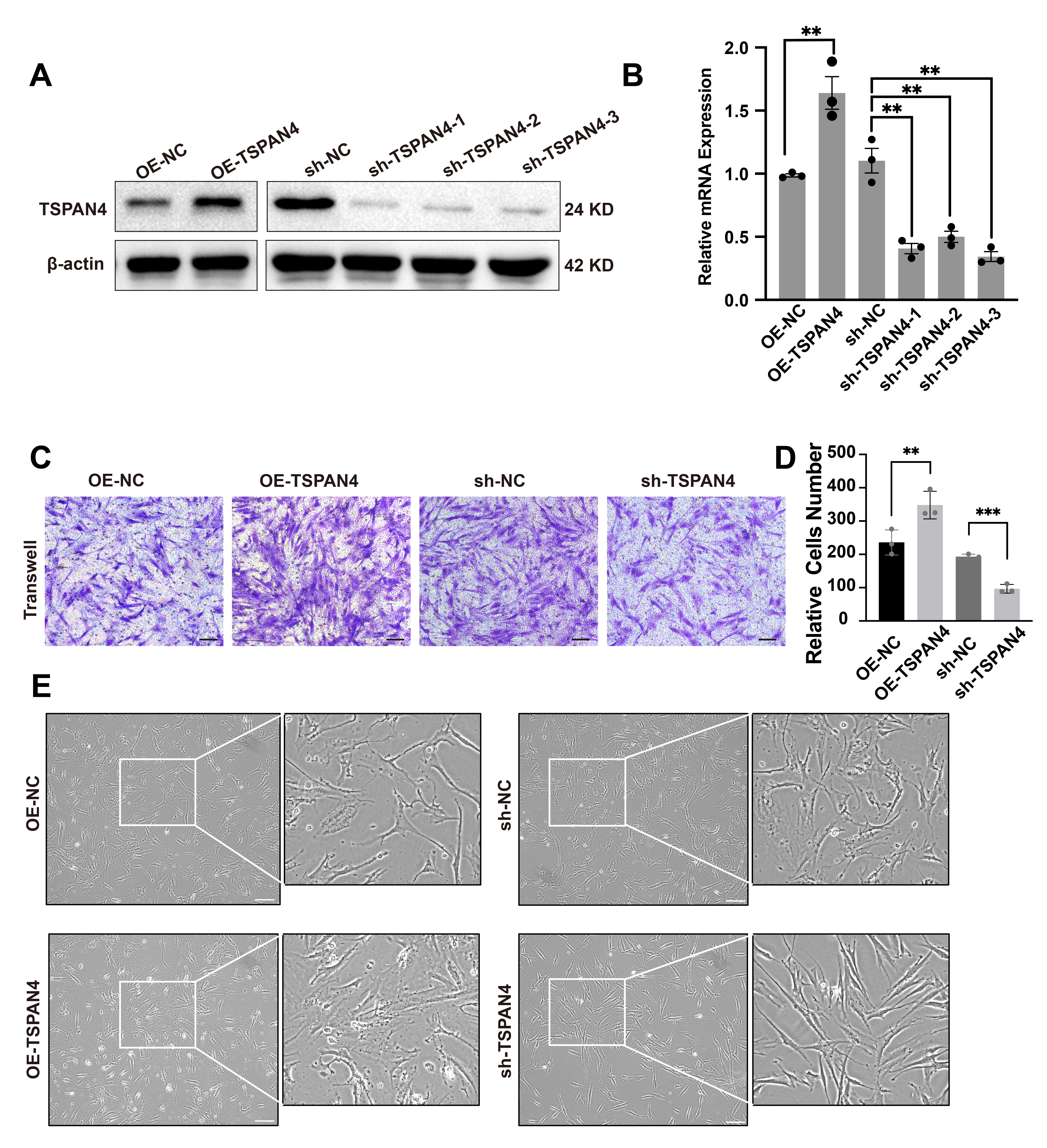

Supplement: Online supplementary figure 2 [file cs-139-19-CS20255833-s002.jpg]

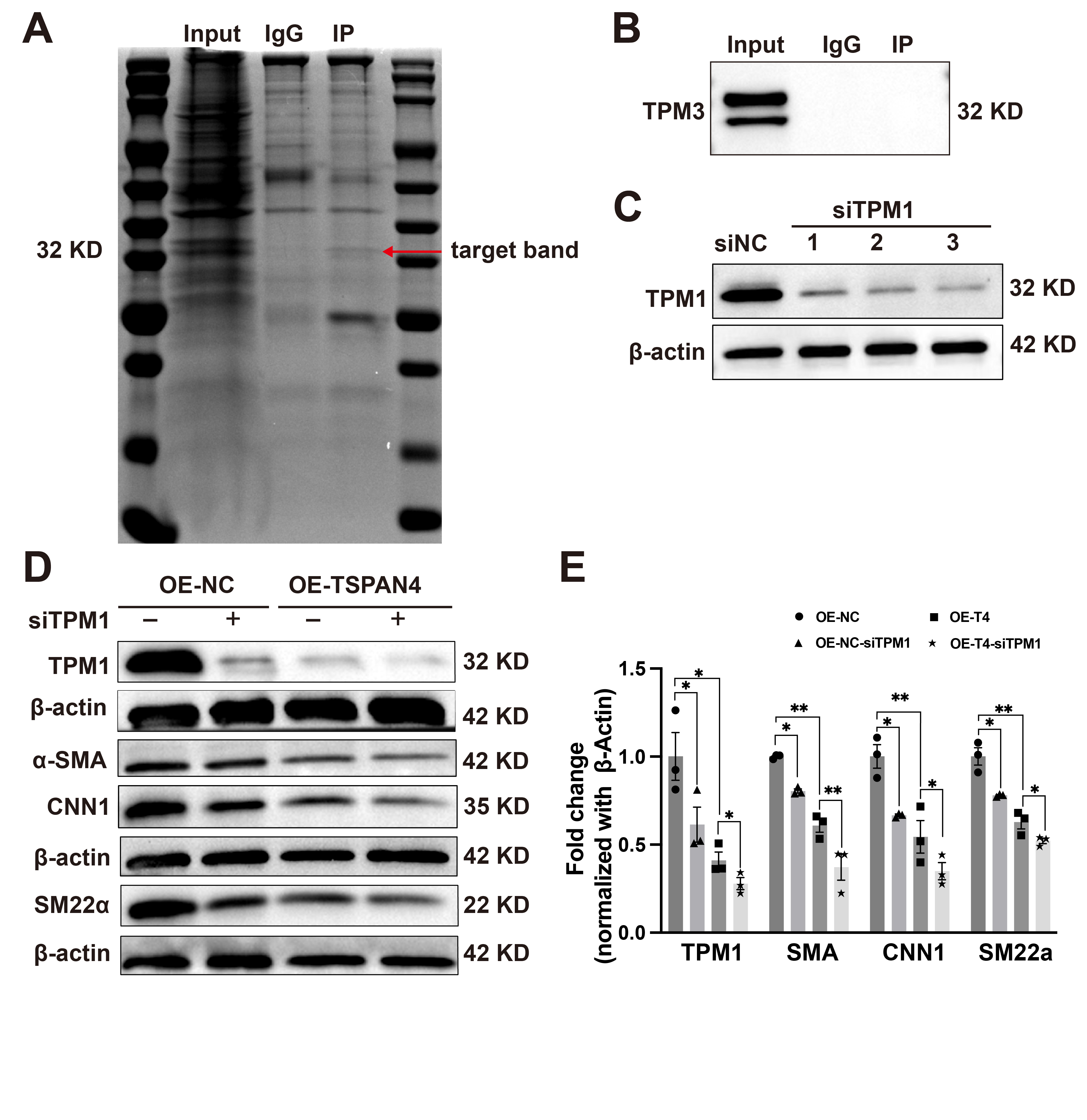

Supplement: Online supplementary figure 3 [file cs-139-19-CS20255833-s003.jpg]

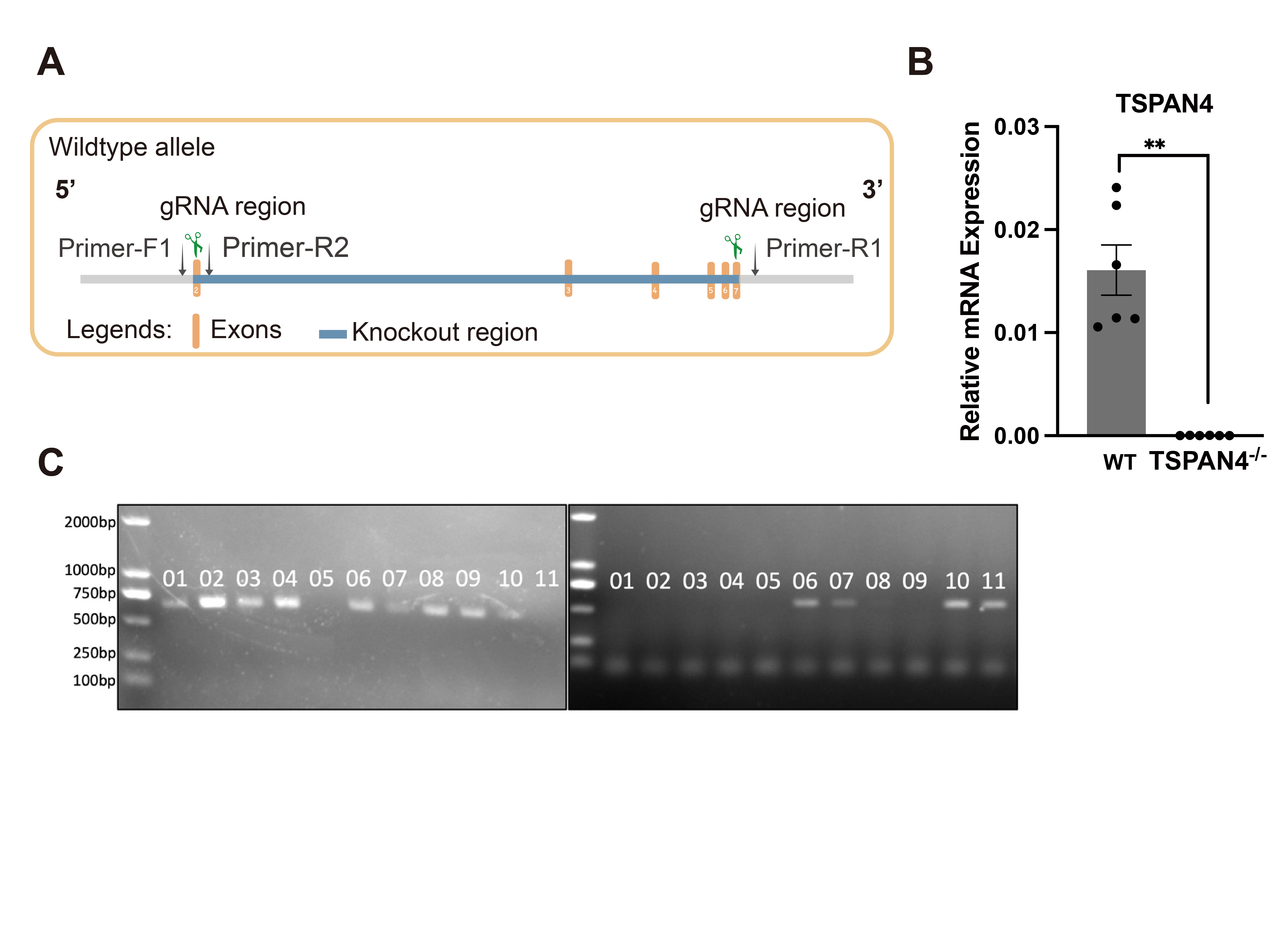

Supplement: Online supplementary figure 4 [file cs-139-19-CS20255833-s004.jpg]
