# Supplementary material for: TSPAN4 controls vascular smooth muscle cell phenotypic switching and intimal hyperplasia by targeting TPM1-regulated cytoskeletal organization
Source: Clin Sci (Lond). 2025 Oct 8;139(19):1145–61. doi: 10.1042/CS20255833 (PMC12687436; doi:10.1042/CS20255833)
Supplement: Online supplementary table 1 [file cs-139-19-CS20255833-s005.docx]

**Supplementary Table 1. List of antibodies used in this study**

| Antibodies | Source | Identifier |
| --- | --- | --- |
| smooth muscle actin specific Monoclonal antibody | Proteintech | 67735-1-Ig |
| transgelin/SM22 Polyclonal antibody | Proteintech | 10493-1-AP |
| Calponin Polyclonal antibody | Proteintech | 13938-1-AP |
| Osteopontin Rabbit Recombinant mAb | Selleck | A5427 |
| TPM1 Polyclonal antibody | Proteintech | 28477-1-AP |
| TPM3 Polyclonal antibody | Proteintech | 10737-1-AP |
| Beta Actin Monoclonal antibody | Proteintech | 66009-1-Ig |
| Tetraspanin-4 Antibody | Novus | NBP1-59438 |
| Polyclonal Rabbit anti‑Human TSPAN4 Antibody | LSBio | LS‑C496962 |
| Alexa Fluor® 488 Donkey Anti-Rabbit IgG (H+L) Antibody | Invitrogen | A21206 |
| Alexa Fluor® 568 Donkey Anti-Rabbit IgG (H+L) Antibody | Invitrogen | A10042 |
| Alexa Fluor® 647 Donkey Anti-Rabbit IgG (H+L) Antibody | Invitrogen | A-31573 |
